# Effects of a Combination of Empagliflozin Plus Metformin vs. Metformin Monotherapy on NAFLD Progression in Type 2 Diabetes: The IMAGIN Pilot Study

**DOI:** 10.3390/biomedicines11020322

**Published:** 2023-01-23

**Authors:** Alfredo Caturano, Raffaele Galiero, Giuseppe Loffredo, Erica Vetrano, Giulia Medicamento, Carlo Acierno, Luca Rinaldi, Aldo Marrone, Teresa Salvatore, Marcellino Monda, Celestino Sardu, Raffaele Marfella, Ferdinando Carlo Sasso

**Affiliations:** 1 Department of Advanced Medical and Surgical Sciences, University of Campania Luigi Vanvitelli, I-80138 Naples, Italy; 2 Department of Precision Medicine, University of Campania Luigi Vanvitelli, I-80138 Naples, Italy

**Keywords:** type 2 diabetes, non-alcoholic fatty liver disease, sodium-glucose cotransporter inhibitors, metformin, FibroScan^®^, controlled attenuation parameter

## Abstract

Non-alcoholic fatty liver disease (NAFLD) comprises a heterogeneous group of metabolic liver diseases and is characterized by the presence of steatosis in at least 5% of hepatocytes. The aim of our study was to assess the effect of the combination therapy of empagliflozin + metformin vs. metformin monotherapy on NAFLD progression in type 2 diabetic (T2DM) patients. Sixty-three metformin-treated T2DM patients who were SGLT2i-naïve and had an ultrasound diagnosis of NAFLD (aged 60.95 ± 11.14 years; males, 57.1%) were included in the present analysis. Thirty-three started the combination therapy. All patients were observed for 6 months and routinely monitored with anthropometry, blood biochemistry, and FibroScan^®^/CAP. At the 6-month follow-up, the combination therapy group presented a significant reduction in BMI (30.83 ± 3.5 vs. 28.48 ± 3.25), glycated hemoglobin (8.2 (7.4–8.8)) vs. 7.2 (6.8–7.9), ALT (68.5 (41.5–88.0) vs. 45.00 (38.00, 48.00)), CAP parameter (293.5 (270.0–319.25) vs. 267.00 (259.50, 283.75)) and steatosis degree (*p* = 0.001) in comparison with the control group, whose parameters remained almost stable over time. In patients affected by T2DM, the combination of empagliflozin + metformin vs. metformin monotherapy ameliorated liver steatosis, ALT levels, body weight, and glycated hemoglobin after a 6-month follow-up.

## 1. Introduction

Non-alcoholic fatty liver disease (NAFLD) comprises a heterogeneous group of metabolic liver diseases, ranging from simple non-alcoholic fatty liver to non-alcoholic steatohepatitis, and is characterized by the presence of steatosis in at least 5% of hepatocytes (demonstrated by imaging or histology) not associated with excessive alcohol consumption or other causes (medications or congenital disorders) [1]. NAFLD has currently reached a prevalence of epidemic proportions, ranging from 20–30% in the general adult population to peaks of over 70% among diabetics [2]. The pathophysiological mechanisms underlying the close relationship between NAFLD and T2DM are multiple (mainly insulin resistance), complex, and only partially known [3] Liver biopsy is the gold standard for NAFLD diagnosis, though it is highly invasive and expensive. Proton density fat fraction calculated by magnetic resonance imaging is the most sensitive and specific non-invasive method for detecting even the slightest degree of hepatic steatosis involving 5–10% of the parenchyma. However, given its scarce availability and high cost, it cannot be used as a screening method. Thus, more economic and less invasive technics have spread and are routinely used in daily practice, such as abdomen ultrasound and the fatty liver index, which makes use of four clinical laboratory parameters (body mass index, waist circumference, serum triglycerides, and serum gamma-glutamyl-transferase). FibroScan^®^, implemented with CAP (controlled attenuation parameter), is another non-invasive and less expensive diagnostic tool that is able to evaluate liver fibrosis by testing its stiffness (rigidity), expressed in kiloPascals (kPa), and quantify the hepatic steatosis with good accuracy [1,4].

The increasing concern about this disease is strictly linked to the rise in liver complications (non-alcoholic steatohepatitis, liver cirrhosis, and hepatocellular carcinoma), increased cardiovascular complications, and overall mortality [5,6,7]. Moreover, there is still uncertainty about the safety of antihyperglycemic treatments in diabetic patients with NAFLD [1,8].

According to most recent guidelines, NAFLD’s only therapeutic option is represented by lifestyle change to promote weight loss through diet and/or physical activity [1]. No pharmacological therapy has been approved for NAFLD treatment. Metformin is not only the first pharmacological line in the treatment of T2DM according to all international scientific guidelines, but it also exerts numerous extra anti-hyperglycaemic ancillary effects, which could soon broaden its therapeutic indications [9,10]. Metformin’s benefits, including inhibiting hepatic gluconeogenesis, modifying hepatic fatty acid metabolism, increasing fatty acid oxidation, reducing lipogenesis, enhancing insulin sensitivity, and increasing antioxidant properties, are well-established [11,12,13]. Several studies have reported that these favorable effects might induce amelioration of liver histology in patients with NAFLD/NASH [11]. In subjects with high CV risk or heart failure and renal damage, the association with sodium–glucose cotransporter-2 inhibitors (SGLT2i) is strongly suggested [14]. Although several hypotheses currently exist, the effects of SGLT2i inhibitors on NAFLD development and/or regression are not fully understood, and further investigations are required [15]. Up to now, only a few observational studies and one randomized control trial on a limited number of patients have suggested SGLT2i’s efficacy in ameliorating liver steatosis with FibroScan^®^/CAP evaluation [16].

The aim of the present pilot study was to compare the effects of a combination of empagliflozin plus metformin vs. metformin monotherapy on liver steatosis using FibroScan^®^/CAP in a cohort of diabetic patients.

## 2. Methods and Materials

### 2.1. Study Population

“Impact of anti-hyperglyceMic Agents on NAFLD proGressIoN in type 2 Diabetes” (IMAGIN) is a single-center prospective observational pilot study. SGLT2i-naïve patients aged ≥18 years with metformin-treated T2DM, an estimated glomerular filtration rate of ≥45 mL/min/m^2^ calculated according to CKD-EPI, and the presence of NAFLD documented by B-mode ultrasound (mild, moderate, or severe bright liver in a patient with no history of hemochromatosis, cystinosis, and/or glycosphingolipidosis of Gaucher type 1) were included in the study. The exclusion criteria were any other form of diabetes; severe cardiac, liver and/or renal insufficiency; an acute cardiovascular event that occurred in the previous 3 months, pregnancy; neoplasm without 6 months of remission; other forms of hepatitis; any inflammatory disease; prolonged use of steroids; and daily alcohol consumption of >20 g (for males) or >10 g (for females). A total of 127 patients with metformin-treated T2DM who were followed at the internal medicine clinic of the University of Campania “Luigi Vanvitelli” were consecutively screened between March 2021 and May 2022. Patients who were not SGLT2i-naïve (*n* = 7), were already undergoing combination therapy (*n* = 30) or insulin therapy (*n* = 7), had severe kidney impairment (*n* = 12), and did not provide written consent (*n* = 8) were excluded. Thus, 63 patients were enrolled in the present study, and follow-up was censored in October 2022. Patients were assigned to a metformin monotherapy group or started the combination therapy of metformin + SGLT2i according to the standards of care of the American Diabetes Association [17].

All patients provided written informed consent for data storage and analysis. The study was conducted in accordance with the Declaration of Helsinki and was approved by the ethics committee of the Azienda Ospedialiera Universitaria of Università degli Studi della Campania “Luigi Vanvitelli” (Ethics Committee Review (2021) No. 0005501).

### 2.2. Procedures

Anthropometric clinical data and blood samples were collected on the same date of the FibroScan^®^ examinations at visit 0 and follow-up (6 months). Blood samples were collected after an 8-h overnight fast and included hemochrome, albumin, transaminases, and glycated hemoglobin.

### 2.3. FibroScan^®^/CAP Measurements

FibroScan^®^ Mini + 430 powered with CAP (Echosens SA, France) was used for the present analysis. CAP and liver stiffness were measured by the same trained operator with an M probe (3.5 MHz), which was placed on the skin in the intercostal space over the right lobe of the liver. Liver stiffness was calculated over at least 10 valid measurements, with a ratio of the interquartile range (IQR) to the median of the liver stiffness (IQR/Median) of ≤30%. Notably, CAP, through the SmartExam program, was continuously computed during the entire examination until the CAP gauge reached 100%. The hepatic steatosis grade was defined by the cut-off values of CAP according to previous reports (sensitivity 82.3%, specificity 55.7% for the 238 dB/m cut-off). CAP < 238 dB/m denoted the absence of steatosis (S0), 238 ≤ CAP ≤ 259 dB/m denoted mild steatosis (grade S1), 260 ≤ CAP ≤ 291 dB/m denoted moderate steatosis (grade S2), and CAP > 291 dB/m denoted severe steatosis (grade S3). ΔCAP was measured by the following formula: (initial CAP—follow-up CAP)/initial CAP. The hepatic fibrosis cut-off value was liver stiffness ≥ 7.0 kPa [18].

### 2.4. Study Endpoint

The primary study endpoint was to assess the effects of a combination of gliflozin plus metformin vs. metformin monotherapy on NAFLD in our cohort of T2DM patients.

### 2.5. Statistical Analysis

Categorical data were expressed as numbers and percentages, whereas continuous variables were expressed as either the median (interquartile range (IQR)) or mean ± SD, depending on their distribution as assessed by the Shapiro–Wilk test. Population data were divided into different groups according to the antidiabetic treatment (metformin of metformin + SGLT2i). Between-group differences for categorical variables were assessed by the chi-square test with the application of Yates correction where appropriate. Either the parametric Student’s t-test or the nonparametric Mann–Whitney U test was used to compare continuous variables, depending on their distribution. All statistical tests for the two subgroups were two-sided and evaluated at the 0.05 level of significance. Finally, we performed a box plot analysis to evaluate the CAP variation between the two subgroups. All statistical analyses were performed through the RStudio^®^ software (RStudio, Boston, MA, USA).

## 3. Results

**Figure 1:**
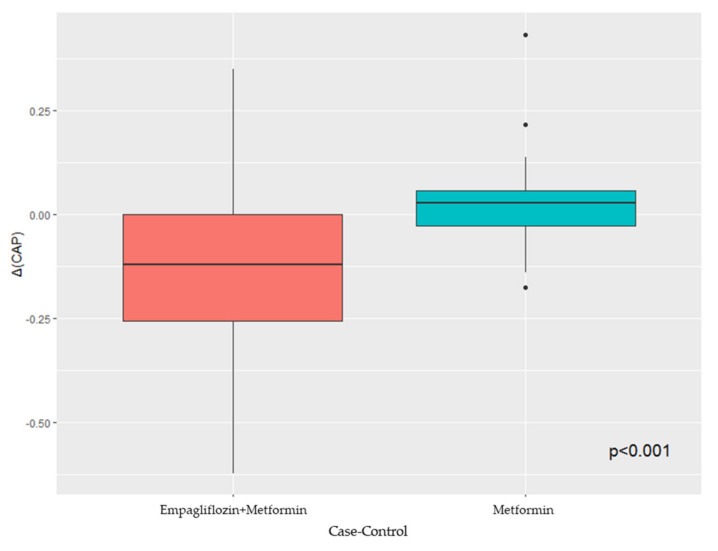
Box plot analysis of CAP variation in case and control groups.

Sixty-three metformin-treated T2DM patients with an echographic diagnosis of NAFLD (aged 60.95 ± 11.14 years; males, 57.1%) were included in the present analysis. Among them, 33 patients continued metformin monotherapy (control group), whilst 30 patients (47.6%) received a combination of metformin + empagliflozin (experimental group). The case group showed a higher level of glycated hemoglobin and AST, and fewer patients were treated with angiotensin-converting enzyme inhibitors (ACEi) and diuretics. All baseline characteristics are reported in Table 1 and Table 2. At the 6-month follow-up, the experimental group presented a significant reduction in BMI, glycated hemoglobin, ALT, CAP parameter, and steatosis degree in comparison with the control group (Table 2). The box plot analysis showed that the median ΔCAP was significantly lower in patients in the experimental group compared with those in the control group (Figure 1).

Of note, no adverse events were reported by our patients during the observation phase.

## 4. Discussion

Among our cohort of metformin-treated T2DM patients, the addition of an SGLT2i proved to reduce ALT levels, body weight, CAP parameter and variation, steatosis degree, and glycaemic control improvement.

SGLT2i exerts its action by selectively inhibiting SGLT2 in the kidneys, leading to an insulin-independent lowering of blood glucose levels (0.6–0.8% glycated hemoglobin reduction) through a boosted daily urinary loss of up to 100 g of glucose (200–300 kcal) and an increased natriuretic effect due to sodium reabsorption inhibition [14,19,20].

Adipose tissue is considered a real endocrine organ capable of producing various biochemical compounds called adipokines, which exert autocrine, paracrine, and endocrine functions by acting on the body’s energetic metabolism [21]. One of the hormones produced by adipose tissue is leptin, which communicates within the liver by activating the JAK-2, STAT-3, MAPK/ERK, and PI3K/AKT/mTOR pathways [22]. At low levels, it exerts a protective effect on hepatic steatosis due to its insulin-sensitizing effects, suppression of gluconeogenesis, and de novo lipogenesis, as well as its stimulation of FFA beta-oxidation [23]. However, leptin overproduction, due to increased adipose tissue, acts as a pro-inflammatory and fibrogenic factor acting on metalloproteinases (TIMP metallopeptidase inhibitor 1 and matrix metalloproteinase-1), stimulating TGF-β, and upregulating CD14 on liver Kupffer cells, thus worsening liver steatosis [24,25,26,27]. SGLT2i clinical trials have reported moderate weight loss caused by a reduction in the volume of both the abdominal visceral adipose tissue and subcutaneous adipose tissue, thus also ameliorating metabolic syndrome. These mechanisms lead to a reduction in insulin resistance, which has been shown to play a central role in the reduction of other important clinical outcomes closely related to metabolic syndrome [28,29,30,31]. In particular, endothelial dysfunction amelioration, together with the reduction in inflammation and reactive oxygen species production as well as metabolic changes, are involved in cardiorenal and hepatic risk reduction [28,29,30,31]. In fact, insulin resistance is involved in both glucose and lipid metabolism and is responsible for increased gluconeogenesis and decreased glucose consumption and re-uptake, as well as increased lipogenesis and decreased lipolysis with an increase in the level of triglyceride accumulation in the liver, due to the FOXO1-mediated pathway [32,33]. Indeed, FOXO1 intensifies the transcription of the sterol regulatory element-binding transcription factor 1c involved in lipogenesis. At the same time, once FOXO1 is translocated out of the nucleus, it hinders the transcription of G6P, triggering an increase in lipogenesis against glycogen production [34].

Since SGLT2i commercialization, increasing evidence has reported the efficacy of this class of drug in several clinical scenarios, with particular emphasis on cardiovascular risk reduction, which is enhanced in T2DM and NAFLD patients regardless of other major risk factors. In animal models, SGLT2i proved to have efficacy in reducing liver steatosis, fibrosis, and inflammation [15]. It has been suggested that these changes might be due to a negative energy balance, which in turn is a result of enhanced glycosuria and a substrate shift toward lipids that might be promoted by an increased glucagon/insulin ratio [35]. These same effects are also responsible for both improved glycaemic control and weight loss, as reported by several studies [35,36]. Due to this, the use of SGLT2i has been considered for the treatment of NAFLD patients. In our study, the addition of the SGLT2i empagliflozin to metformin induced a statistically significant reduction in the median CAP parameter of 9% (3.9–11.1%). Similar findings were reported by a Japanese open-label trial on 57 patients with T2DM and NAFLD randomized to 5 mg dapagliflozin per day for 24 weeks (*n* = 33) or to a control group (*n* = 24). They reported a CAP parameter reduction from a baseline of 315 ± 61 dB/m to 290 ± 73 dB/m at follow-up [37]. By contrast, the only randomized control trial published, which enrolled 44 patients with T2DM and NAFLD who were randomized to receive 50 mg/day of ipragliflozin as an add-on treatment (*n* = 29) or continued metformin and pioglitazone (*n* = 15) for 24 weeks, exhibited a non-significant CAP decline in the intervention group [16]. However, the authors reported improvement in NAFLD parameters and serum ALT levels, similar to our results, and this has been suggested to be associated with reductions in body weight and HbA1c levels [16,38]. Moreover, in a prospective randomized, double-blind, placebo-controlled trial enrolling 106 patients with both NAFLD and T2DM who were assigned to receive empagliflozin 10 mg (*n* = 35), pioglitazone 30 mg (*n* = 34), or placebo (*n* = 37) for 24 weeks, only a borderline significant decrease in CAP score was observed with empagliflozin compared with placebo (−29.6 dB/m (−39.5 to −19.6) vs. −16.4 dB/m (−25.0 to −7.8), respectively; *p* = 0.05) [39].

Liver fat content amelioration associated with SGLT2is treatment has also been assessed through magnetic resonance imaging. A potentially consistent reduction in liver fat, especially when ALT levels are high, was suggested by the analysis of several datasets collected in individuals with T2DM, including the EMPA-REG OUTCOME^®^ trial. However, liver fat was not directly measured in any of the studies included in these analyses, and only one study using magnetic resonance spectroscopy showed that ALT levels reasonably correlated with liver fat in individuals with T2DM (*r* = 0.66) [40]. In a recent meta-analysis of 12 randomized controlled trials estimating the efficacy of different SGLT2is on NAFLD and enrolling 850 overweight or obese individuals followed for 24 weeks, a decrease in ALT levels as well as fatty liver content was reported [28]. These conclusions were also confirmed by another larger meta-analysis, enrolling 1950 patients with T2DM with and without NAFLD who were treated with SGLT2i for at least 8 weeks and 1900 control patients [41]. In the E-LIFT trial, 50 patients with type 2 diabetes and NAFLD were enrolled and randomized to an empagliflozin group (standard treatment plus empagliflozin 10 mg daily) or a control group (standard treatment without empagliflozin). After 20 weeks, they reported a significant reduction in liver fat content according to magnetic resonance imaging (about 4.9%) and reduced ALT levels [42].

Eight patients enrolled in our study had liver stiffness of severe grade of fibrosis according to the FibroScan^®^ evaluations. At follow-up, we assisted in a reduction in patients’ median stiffness value, though this was not statistically significant. It seems possible that this may be due to the reduced glucotoxicity secondary to better glycaemic control and the reduced liver fat accumulation and inflammation. Moreover, it is also possible that several patients could have been affected by undiagnosed NASH. Cumulative evidence in both preclinical and clinical scenarios has proven SGLT2i’s efficacy in NASH resolution. It is possible that, beyond the aforementioned mechanisms, SGLT2i could have played a role in reducing stiffness parameters, though more studies are needed [15,43]. FIB-4 evaluation, instead, showed a numerical increase, though it was not significant. This may be due to the mathematical formula used to calculate FIB-4. In fact, the ALT levels, which were reduced during treatment, are reported in the denominator, thus increasing their value. In any case, the mean value reported was not significant for fibrosis [44].

## 5. Limitations

Our study presents some limitations. First of all, this is a single-center pilot study with a small sample size, thus limiting the generalization of our results. Moreover, dietary habits, which are potential confounders, were not documented systematically during the study. However, none of our patients reported changes in their lifestyles regarding dietary habits or physical exercise. Finally, this prospective observational study does not allow us to define a cause–effect relationship but only an association between metformin plus empagliflozin combination therapy and an improvement in the assessed endpoints.

## 6. Conclusions

In T2DM patients, the combination of empagliflozin + metformin vs. metformin monotherapy ameliorated liver steatosis (with amelioration of 9% of the CAP value), ALT levels, body weight, and glycated haehemoglobinter only a 6-month follow-up. The promising preliminary results of this pilot study need to be confirmed in a larger population and above all by ad hoc-designed RCTs.

## Figures and Tables

**Table 1:** Baseline clinical characteristics of the study sample.

BASELINE				
Parameter	Overall (*n* = 63)	Control Group (*n* = 33)	Combination Group (*n* = 30)	*p*-Value
**Age**, mean (SD)	60.95 (11.14)	60.09 (11.47)	61.90 (10.88)	0.524
**Sex**, *n* (%)				
*Male*	36 (57.1)	19 (57.6)	17 (56.7)	1.000
*Female*	27 (42.9)	14 (42.4)	13 (43.3)	
**Hypertension**, *n* (%)	43 (68.3)	21 (63.6)	22 (73.3)	0.579
**Smoking habit**, *n* (%)	26 (41.3)	13 (39.4)	13 (43.3)	0.951
**ACEi**, *n* (%)	12 (19.0)	10 (30.3)	2 (6.7)	**0.039**
**ARB**, *n* (%)	12 (19.0)	9 (27.3)	3 (10.0)	0.155
**Diuretic**, *n* (%)	6 (9.5)	6 (18.2)	-	**0.043**
**Beta-blockers**, *n* (%)	8 (12.7)	6 (18.2)	2 (6.7)	0.321
**Alfa blockers**, *n* (%)	1 (1.6)	-	1 (3.3)	0.962
**Ca antagonist**, *n* (%)	4 (6.3)	2 (6.1)	2 (6.7)	1.000
**Statin**, *n* (%)	31 (49.2)	15 (45.5)	16 (53.3)	0.710

SD, standard deviation; ACEi, angiotensin-converting enzyme inhibitor; ARB, angiotensin receptor blocker; Ca, calcium.

**Table 2:** Comparison of baseline and follow-up clinical, anthropometric, and laboratory data of the study sample.

PROGRESSION TIME						
	Time 0 (Baseline)			Time 1 (=6 months)		
Parameter	Control Group (*n* = 33)	Combination Group (*n* = 30)	*p*-Value	Control Group (*n* = 33)	Combination Group (*n* = 30)	*p*-Value
**Bmi**, mean (SD)	31.89 (4.65)	30.83 (3.52)	0.314	31.91 (4.66)	28.48 (3.25)	**0.023**
**HbA1_c_**, median [IQR]	7.10 [6.50, 7.50]	8.20 [7.40, 8.80]	**0.002**	7.20 [6.50, 7.50]	7.20 [6.80, 7.90]	0.552
**Ast**, median [IQR]	38.00 [26.00, 42.00]	44.00 [28.50, 47.75]	0.139	38.00 [27.00, 44.00]	41.00 [25.00, 46.00]	0.789
**Alt**, median [IQR]	49.00 [32.00, 67.00]	68.5 [41.50, 88.00]	**0.050**	56.00 [43.00, 72.00]	45.00 [38.00, 48.00]	**0.006**
**Platelet**, median [IQR]	210000 [143000, 243000]	236500 [193250, 287500]	0.054	221000 [145000, 242000]	234000 [199000, 250000]	0.253
**Albumin**, median [IQR]	4.10 [3.80, 4.32]	4.00 [3.80, 4.40]	0.873	4.20 [4.00, 4.30]	4.16 [3.98, 4.40]	0.911
**Stiffness kPa**, median [IQR]	7.20 [5.70, 10.40]	8.30 [6.42, 10.15]	0.248	7.40 [6.00, 10.62]	6.80 [5.55, 8.10]	0.237
**Degree Fibrosis**, *n* (%)						
0–1	14 (42.4)	9 (30.0)	0.566	11 (33.3)	17 (56.7)	0.151
2	10 (30.3)	12 (40.0)		12 (36.4)	9 (30.0)	
3	6 (18.2)	4 (13.3)		6 (18.2)	1 (3.3)	
4	3 (9.1)	5 (16.7)		4 (12.1)	3 (10.0)	
**CAP**, median [IQR]	280.00 [258.00, 310.00]	293.50 [270.00, 319.25]	0.401	289.00 [259.00, 324.00]	267.00 [259.50, 283.75]	**0.036**
**Degree Steatosis**, *n* (%)						
0	4 (12.1)	2 (6.7)	0.382	6 (18.2)	3 (10.0)	**0.001**
1	6 (18.2)	2 (6.7)		3 (9.1)	5 (16.7)	
2	7 (21.2)	10 (33.3)		9 (27.3)	20 (66.7)	
3	16 (48.5)	16 (53.3)		15 (45.5)	2 (6.7)	
**FIB-4**, median [IQR]	1.33 [0.94, 1.84]	1.39 [1.08, 1.68]	0.934	1.42 [1.13, 1.55]	1.58 [1.35, 1.87]	0.174

SD, standard deviation; BMI, body mass index; HbA1_c_, glycated hemoglobin; IQR, interquartile range; AST, aspartate aminotransferase; ALT, alanine aminotransferase; CAP, controlled attenuated parameter; FIB-4, fibrosis-4.

## Data Availability

The data that support the findings of this study are available upon reasonable request from the corresponding author.

## References

[1] European Association for the Study of the Liver (EASL), European Association for the Study of Diabetes (EASD), European Association for the Study of Obesity (EASO) (2016). EASL-EASD-EASO Clinical Practice Guidelines for the management of non-alcoholic fatty liver disease. J. Hepatol..

[2] Rinaldi L., Pafundi P.C., Galiero R., Caturano A., Morone M.V., Silvestri C., Giordano M., Salvatore T., Sasso F.C. (2021). Mechanisms of Non-Alcoholic Fatty Liver Disease in the Metabolic Syndrome. A Narrative Review. Antioxidants.

[3] Acierno C., Caturano A., Pafundi P.C., Nevola R., Adinolfi L.E., Sasso F.C. (2020). Nonalcoholic fatty liver disease and type 2 diabetes: Pathophysiological mechanisms shared between the two faces of the same coin. Explor. Med..

[4] Sasso M., Tengher-Barna I., Ziol M., Miette V., Fournier C., Sandrin L., Poupon R., Cardoso A.C., Marcellin P., Douvin C. (2012). Novel controlled attenuation parameter for noninvasive assessment of steatosis using Fibroscan(^®^): Validation in chronic hepatitis C. J. Viral Hepat..

[5] Galiero R., Caturano A., Vetrano E., Cesaro A., Rinaldi L., Salvatore T., Marfella R., Sardu C., Moscarella E., Gragnano F. (2021). Pathophysiological mechanisms and clinical evidence of relationship between Nonalcoholic fatty liver disease (NAFLD) and cardiovascular disease. Rev. Cardiovasc. Med..

[6] Caturano A., Acierno C., Nevola R., Pafundi P.C., Galiero R., Rinaldi L., Salvatore T., Adinolfi L.E., Sasso F.C. (2021). Non-Alcoholic Fatty Liver Disease: From Pathogenesis to Clinical Impact. Processes.

[7] Sasso F.C., Carbonara O., Cozzolino D., Rambaldi P., Mansi L., Torella D., Gentile S., Turco S., Torella R., Salvatore T. (2000). Effects of insulin-glucose infusion on left ventricular function at rest and during dynamic exercise in healthy subjects and noninsulin dependent diabetic patients: A radionuclide ventriculographic study. J. Am. Coll. Cardiol..

[8] Gentile S., Turco S., Guarino G., Oliviero B., Annunziata S., Cozzolino D., Sasso F.C., Turco A., Salvatore T., Torella R. (2001). Effect of treatment with acarbose and insulin in patients with non-insulin-dependent diabetes mellitus associated with non-alcoholic liver cirrhosis. Diabetes Obes. Metab..

[9] Salvatore T., Pafundi P.C., Morgillo F., Di Liello R., Galiero R., Nevola R., Marfella R., Monaco L., Rinaldi L., Adinolfi L.E. (2020). Metformin: An old drug against old age and associated morbidities. Diabetes Res. Clin. Pract..

[10] Salvatore T., Pafundi P.C., Galiero R., Rinaldi L., Caturano A., Vetrano E., Aprea C., Albanese G., Di Martino A., Ricozzi C. (2020). Can Metformin Exert as an Active Drug on Endothelial Dysfunction in Diabetic Subjects?. Biomedicines.

[11] Zhou J., Massey S., Story D., Li L. (2018). Metformin: An Old Drug with New Applications. Int. J. Mol. Sci..

[12] Di Francia R., Rinaldi L., Troisi A., Di Benedetto F., Berretta M. (2015). Effect of anti-oxidant agents in patients with hepatocellular diseases. Eur. Rev. Med. Pharmacol. Sci..

[13] Di Francia R., Rinaldi L., Cillo M., Varriale E., Facchini G., D’Aniello C., Marotta G., Berretta M. (2016). Antioxidant diet and genotyping as tools for the prevention of liver disease. Eur. Rev. Med. Pharmacol. Sci..

[14] Draznin B., Aroda V.R., Bakris G., Benson G., Brown F.M., Freeman R., Green J., Huang E., Isaacs D., American Diabetes Association Professional Practice Committee (2022). 9. Pharmacologic Approaches to Glycemic Treatment: Standards of Medical Care in Diabetes-2022. Diabetes Care.

[15] Katsiki N., Perakakis N., Mantzoros C. (2019). Effects of sodium-glucose co-transporter-2 (SGLT2) inhibitors on non-alcoholic fatty liver disease/non-alcoholic steatohepatitis: Ex quo et quo vadimus?. Metabolism.

[16] Han E., Lee Y.H., Lee B.W., Kang E.S., Cha B.S. (2020). Ipragliflozin Additively Ameliorates Non-Alcoholic Fatty Liver Disease in Patients with Type 2 Diabetes Controlled with Metformin and Pioglitazone: A 24-Week Randomized Controlled Trial. J. Clin. Med..

[17] American Diabetes Association (2021). 9. Pharmacologic Approaches to Glycemic Treatment: Standards of Medical Care in Diabetes-2021. Diabetes Care.

[18] Huang Z., Ng K., Chen H., Deng W., Li Y. (2022). Validation of Controlled Attenuation Parameter Measured by FibroScan as a Novel Surrogate Marker for the Evaluation of Metabolic Derangement. Front. Endocrinol..

[19] Saisho Y. (2020). SGLT2 Inhibitors: The Star in the Treatment of Type 2 Diabetes?. Diseases.

[20] Salvatore T., Pafundi P.C., Galiero R., Albanese G., Di Martino A., Caturano A., Vetrano E., Rinaldi L., Sasso F.C. (2021). The Diabetic Cardiomyopathy: The Contributing Pathophysiological Mechanisms. Front. Med..

[21] Polyzos S.A., Mantzoros C.S. (2015). Leptin in health and disease: Facts and expectations at its twentieth anniversary. Metabolism.

[22] Robertson S.A., Leinninger G.M., Myers M.G. (2008). Molecular and neural mediators of leptin action. Physiol. Behav..

[23] Polyzos S.A., Kountouras J., Mantzoros C.S. (2015). Leptin in nonalcoholic fatty liver disease: A narrative review. Metabolism.

[24] Aleffi S., Petrai I., Bertolani C., Parola M., Colombatto S., Novo E., Vizzutti F., Anania F.A., Milani S., Rombouts K. (2005). Upregulation of proinflammatory and proangiogenic cytokines by leptin in human hepatic stellate cells. Hepatology.

[25] Yan K., Deng X., Zhai X., Zhou M., Jia X., Luo L., Niu M., Zhu H., Qiang H., Zhou Y. (2012). p38 mitogen-activated protein kinase and liver X receptor-α mediate the leptin effect on sterol regulatory element binding protein-1c expression in hepatic stellate cells. Mol. Med..

[26] Cao Q., Mak K.M., Ren C., Lieber C.S. (2004). Leptin stimulates tissue inhibitor of metalloproteinase-1 in human hepatic stellate cells: Respective roles of the JAK/STAT and JAK-mediated H2O2-dependant MAPK pathways. J. Biol. Chem..

[27] Ikejima K., Takei Y., Honda H., Hirose M., Yoshikawa M., Zhang Y.J., Lang T., Fukuda T., Yamashina S., Kitamura T. (2002). Leptin receptor-mediated signaling regulates hepatic fibrogenesis and remodeling of extracellular matrix in the rat. Gastroenterology.

[28] Mantovani A., Petracca G., Csermely A., Beatrice G., Targher G. (2020). Sodium-Glucose Cotransporter-2 Inhibitors for Treatment of Nonalcoholic Fatty Liver Disease: A Meta-Analysis of Randomized Controlled Trials. Metabolism.

[29] Salvatore T., Caturano A., Galiero R., Di Martino A., Albanese G., Vetrano E., Sardu C., Marfella R., Rinaldi L., Sasso F.C. (2021). Cardiovascular Benefits from Gliflozins: Effects on Endothelial Function. Biomedicines.

[30] Sasso F.C., Pafundi P.C., Caturano A., Galiero R., Vetrano E., Nevola R., Petta S., Fracanzani A.L., Coppola C., Di Marco V. (2021). Impact of direct acting antivirals (DAAs) on cardiovascular events in HCV cohort with pre-diabetes. Nutr. Metab. Cardiovasc. Dis..

[31] Adinolfi L.E., Petta S., Fracanzani A.L., Nevola R., Coppola C., Narciso V., Rinaldi L., Calvaruso V., Pafundi P.C., Lombardi R. (2020). Reduced incidence of type 2 diabetes in patients with chronic hepatitis C virus infection cleared by direct-acting antiviral therapy: A prospective study. Diabetes Obes. Metab..

[32] Haeusler R.A., Hartil K., Vaitheesvaran B., Arrieta-Cruz I., Knight C.M., Cook J.R., Kammoun H.L., Febbraio M.A., Gutierrez-Juarez R., Kurland I.J. (2014). Integrated control of hepatic lipogenesis versus glucose production requires FoxO transcription factors. Nat. Commun..

[33] Chen L., Chen X.W., Huang X., Song B.L., Wang Y., Wang Y. (2019). Regulation of glucose and lipid metabolism in health and disease. Sci. China Life Sci..

[34] Choi D.H., Jung C.H., Mok J.O., Kim C.H., Kang S.K., Kim B.Y. (2018). Effect of dapagliflozin on alanine aminotransferase improvement in type 2 diabetes mellitus with non-alcoholic fatty liver disease. Endocr. Metab..

[35] Gunhan H.G., Imre E., Erel P., Ustay O. (2020). Empagliflozin is more effective in reducing microalbuminuria and alt levels compared with dapagliflozin: Real life experience. Acta Endocrinol..

[36] Salvatore T., Galiero R., Caturano A., Rinaldi L., Di Martino A., Albanese G., Di Salvo J., Epifani R., Marfella R., Docimo G. (2022). An Overview of the Cardiorenal Protective Mechanisms of SGLT2 Inhibitors. Int. J. Mol. Sci..

[37] Shimizu M., Suzuki K., Kato K., Jojima T., Iijima T., Murohisa T., Iijima M., Takekawa H., Usui I., Hiraishi H. (2019). Evaluation of the effects of dapagliflozin, a sodium-glucose co-transporter-2 inhibitor, on hepatic steatosis and fibrosis using transient elastography in patients with type 2 diabetes and non-alcoholic fatty liver disease. Diabetes Obes. Metab..

[38] Leiter L.A., Forst T., Polidori D., Balis D.A., Xie J., Sha S. (2016). Effect of canagliflozin on liver function tests in patients with type 2 diabetes. Diabetes Metab..

[39] Chehrehgosha H., Sohrabi M.R., Ismail-Beigi F., Malek M., Reza Babaei M., Zamani F., Ajdarkosh H., Khoonsari M., Fallah A.E., Khamseh M.E. (2021). Empagliflozin Improves Liver Steatosis and Fibrosis in Patients with Non-Alcoholic Fatty Liver Disease and Type 2 Diabetes: A Randomized, Double-Blind, Placebo-Controlled Clinical Trial. Diabetes Ther..

[40] Sattar N., Fitchett D., Hantel S., George J.T., Zinman B. (2018). Empagliflozin is associated with improvements in liver enzymes potentially consistent with reductions in liver fat: Results from randomised trials including the EMPA-REG OUTCOME^®^ trial. Diabetologia.

[41] Coelho F.D.S., Borges-Canha M., von Hafe M., Neves J.S., Vale C., Leite A.R., Carvalho D., Leite-Moreira A. (2021). Effects of sodium-glucose co-transporter 2 inhibitors on liver parameters and steatosis: A meta-analysis of randomized clinical trials. Diabetes Metab. Res Rev..

[42] Kuchay M.S., Krishan S., Mishra S.K., Farooqui K.J., Singh M.K., Wasir J.S., Bansal B., Kaur P., Jevalikar G., Gill H.K. (2018). Effect of Empagliflozin on Liver Fat in Patients With Type 2 Diabetes and Nonalcoholic Fatty Liver Disease: A Randomized Controlled Trial (E-LIFT Trial). Diabetes Care.

[43] Eriksson J.W., Lundkvist P., Jansson P.A., Johansson L., Kvarnström M., Moris L., Miliotis T., Forsberg G.B., Risérus U., Lind L. (2018). Effects of dapagliflozin and n-3 carboxylic acids on non-alcoholic fatty liver disease in people with type 2 diabetes: A double-blind randomised placebo-controlled study. Diabetologia.

[44] Sterling R.K., Lissen E., Clumeck N., Sola R., Correa M.C., Montaner J., Sulkowski M., Torriani F.J., Dieterich D.T., APRICOT Clinical Investigators (2006). Development of a simple noninvasive index to predict significant fibrosis in patients with HIV/HCV coinfection. Hepatology.

